# Glutamate Receptors and Synaptic Plasticity in Health and Disease: A Personal Journey

**DOI:** 10.1002/hipo.70062

**Published:** 2026-01-18

**Authors:** Graham L. Collingridge

**Affiliations:** ^1^ Lunenfeld‐Tanenbaum Research Institute Mount Sinai Hospital Toronto Ontario Canada; ^2^ Tanz Centre for Research in Neurodegenerative Diseases, Department of Physiology, Temerty Faculty of Medicine University of Toronto Toronto Ontario Canada

## Abstract

I describe my progress in understanding synaptic plasticity in the hippocampus. Over the decades my lab has focused on the roles of glutamate receptors (AMPARs, NMDARs, mGluRs and KARs) and associated signaling molecules in LTP and LTD. Most of our studies have been conducted in area CA1 (Schaffer collateral—commissural pathway) with some conducted in CA3 (mossy fiber pathway). We have made extensive use of electrophysiology and pharmacological tools, complemented with knock‐out (KO) and transgenic mice, biochemistry and dynamic imaging. From a starting point in 1980, with essentially no molecular insights available, we have developed a detailed, but still incomplete, mechanism for LTP at CA1 and CA3 synapses as well as providing insights into LTD at CA1 synapses. We have also explored how dysregulated synaptic plasticity contributes to brain disorders, with an emphasis on Alzheimer's disease. Indeed, through a molecular understanding of synaptic plasticity, now we can explain how plaques and tangles are related mechanistically and, in essence, how the early stages of dementia are triggered. Therapeutic strategies, both pharmacological and lifestyle, for tackling dementia are touched upon. Our work, together with that of many other groups, has resulted in massive progress in the understanding of synaptic plasticity in the mammalian CNS in health and disease.

## 1 Introduction

In keeping with the purpose of this collection of papers, here I describe the work that my laboratory has conducted over several decades to try to establish the roles of glutamate receptors, and some associated signaling molecules, in synaptic plasticity in the hippocampus. There is not sufficient space to discuss the extensive body of literature on this topic. Readers interested in a more comprehensive and balanced account of this topic are referred to a recent chapter in The Hippocampus Book (2nd Edition) (Bliss et al. 2024).

**FIGURE 1:**
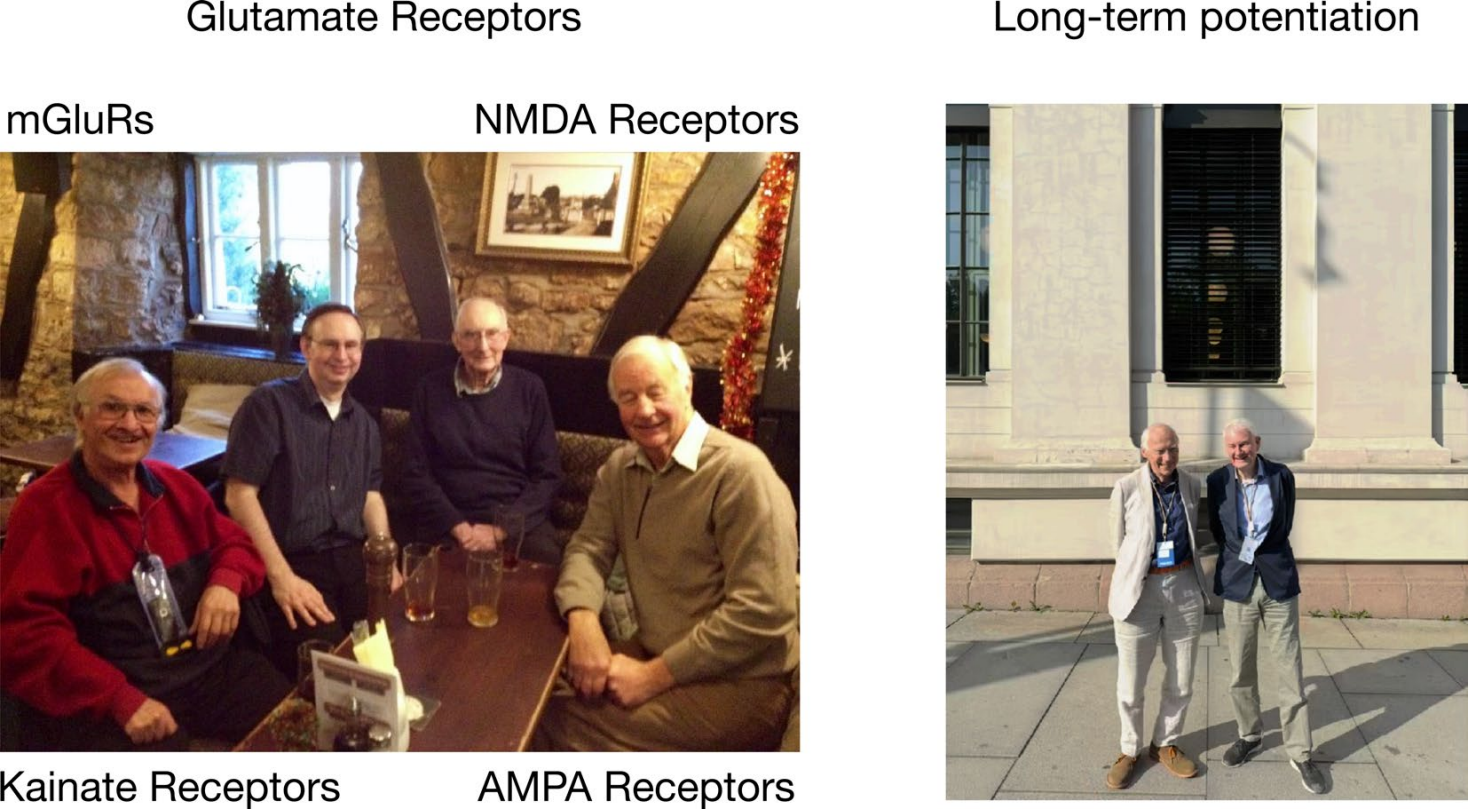
Pioneers of glutamate receptors and LTP. (Left) From left to right: Dick Evans, David Jane, Jeff Watkins, David Lodge. Picture taken in 2014 near Bristol, UK. Each individual contributed to the identification of the four major glutamate receptor classes and their subtypes and the development of key pharmacological reagents. (Right) Tim Bliss and Terje Lomo, the discoverers of LTP. The photograph was taken in 2025 outside of the University of Oslo building where the original experiments were conducted in 1968 and 1969. Both photographs were taken by myself.

My interest in neuroscience began as an undergraduate in the Department of Pharmacology at the University of Bristol in the UK. Here I was exposed to lectures from the late Dick Evans (Collingridge et al. 2024) and the late Jeff Watkins (Collingridge et al. 2023), the father of the glutamate receptor field (Collingridge and Abraham 2022). My interest in the area developed as I studied for a PhD with the late John Davies in London (School of Pharmacy, now UCL) where my first project was to evaluate new NMDAR antagonists that had been synthesized by Jeff Watkins (Collingridge and Davies 1979). My interest in glutamate receptors was reinforced during my first postdoc with the late Hugh McLennan (Department of Physiology, University of British Columbia, Vancouver). These four pioneers in the field of glutamate receptors, together with David Lodge and David Jane (who appears later in my story), are amongst the principal scientists that identified, and developed crucial pharmacological tools for, the major ionotropic glutamate receptors: AMPARs (alpha‐amino‐3‐hydroxy‐5‐methyl‐4‐isoxazolepropionate) receptors, NMDARs (N‐methyl‐D‐aspartate receptors), KARs (kainate receptors), and mGluRs (metabotropic glutamate receptors). These scientists remain my inspiration. Four of my good friends and glutamate receptor pioneers are shown in Figure 1.

I arrived in Vancouver in October 1980 to start my first 2‐year postdoctoral position, where I witnessed an example of long‐term potentiation (LTP), and I was instantly hooked. LTP was discovered in Oslo in the 1960's by Terje Lomo and Tim Bliss, and the history of this fundamental discovery is described in other papers in this collection (Bliss 2025; Lomo 2025). A recent photograph I took of Bliss and Lomo outside of the laboratory in Oslo where LTP was discovered is shown in Figure 1. David West, a visitor to the McLennan laboratory, demonstrated LTP to me using an interface type of recording chamber (see Alger 2025), and I continued with this methodology throughout my time in Vancouver. This brief exposure to a physiological process made very long‐term changes in my brain that persist to this day. One might say that I have become addicted to LTP. Shortly after assuming my first faculty position in the Department of Pharmacology at the University of Bristol in September 1983, I met Tim Bliss (who was based at the National Institute for Medical Research); we instantly became and remain to this day great friends (and occasional co‐authors on review articles) (e.g., Bliss and Collingridge 1993). I firmly believe that the discovery of L‐glutamate as the major neurotransmitter in the vertebrate CNS (principally by Curtis and Watkins) and the discovery of LTP by Bliss and Lomo are two of the very most impactful discoveries in neuroscience (a view supported by citation levels). My major contribution to neuroscience was to be one of the very first to recognize that these two monumental discoveries were closely interrelated—they converge at the level of the synapse. To understand the role of glutamate receptors in synaptic plasticity has been my research mission ever since.

## 2 Vancouver (1980–1982)

I teamed up with Steven Kehl, a graduate student in the McLennan lab, and we set out to explore the roles of glutamate receptors in LTP. Steve verified the selectivity of glutamate antagonists, including D‐AP5 that was a gift from Jeff Watkins, on single hippocampal CA1 neurons (see Box 1) (Collingridge et al. 1983a). Meanwhile I used these pharmacological tools to investigate the synaptic function of glutamate receptors (Collingridge et al. 1983b). It was during this time that I discovered the role of the NMDAR in LTP. In the summer of 1981, I visited Jeff Watkins in Bristol, and he gave me samples of N‐methyl‐D‐aspartic acid (NMDA) and his newly synthesized antagonist, 2‐amino‐5‐phosphonopentanoic acid (AP5/APV). Later that summer, shortly following my return to Vancouver, I remember walking to the Faculty club for a beer after the first clear‐cut result using AP5, thinking that this was pretty cool. Steve and I attended a meeting of The Physiological Society in Aberdeen later that year to communicate our findings. As excited as I was about our work, I had no idea how impactful this result was to become; thousands of publications have since investigated NMDAR‐mediated LTP, frequently using AP5 (and its active *S*‐isomer, commonly referred to as D‐AP5) to block its induction.

## 3 Bristol (1983–1990)

**FIGURE 2:**
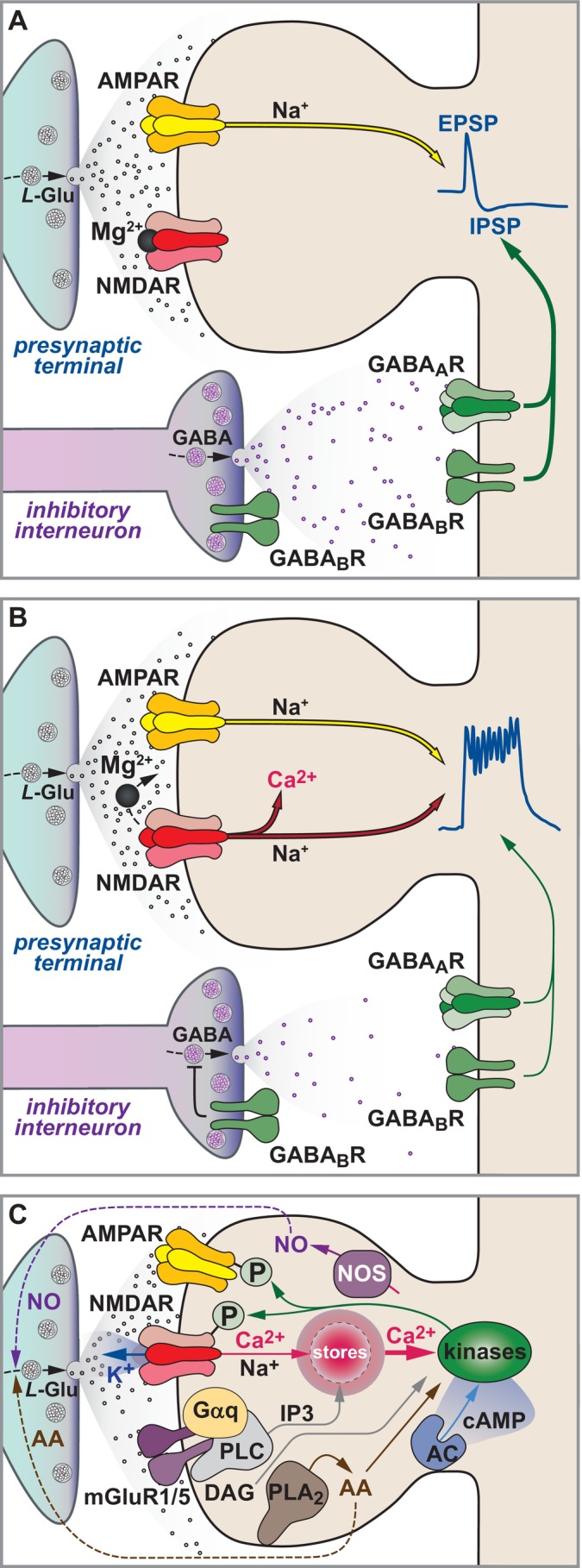
The induction and expression of LTP as seen in the early 1990s. (A) During low frequency stimulation glutamate acts on AMPARs to mediate fast synaptic transmission. The activation of GABAergic neurons quickly hyperpolarizes the membrane to intensify the Mg^2+^ block of NMDARs (Collingridge 2025). (B) During high frequency stimulation the Mg^2+^ block of NMDARs is reduced by depolarization, in part by the temporal summation of AMPAR‐mediated EPSPs (Herron et al. 1986). This is enabled by the activity‐dependent reduction in GABAergic inhibition, orchestrated by presynaptic GABAB receptors (Davies et al. 1991). This mechanism explains, at least in part, the properties of input‐specificity, cooperativity, associativity, the Hebbian nature of synaptic plasticity and the effectiveness of theta burst patterns of stimulation. (C) The expression of LTP. Expression is, in part, due to alterations in AMPAR number/function, involving their phosphorylation. It is also expressed in part by increased L‐glutamate release and may involve retrograde signaling to relay postsynaptic induction to presynaptic expression—three potential retrograde messengers being arachidonic acid (AA), nitric oxide (NO) and potassium ions (K^+^). NMDARs are permeable to Ca^2+^ and there is a magnification of the Ca^2+^ signal by release from intracellular stores. Activation of mGluRs also contributes to the induction of LTP. Several kinases are involved in the process. See (Bliss and Collingridge 1993), from which this figure is adapted and where fuller details and references are available. (At the time of writing, this review had been cited over 15,000 times, according to Google Scholar; reflecting the level of interest in LTP amongst the scientific community).

When I started my own laboratory in Bristol, in 1983, I focused our efforts on trying to understand more about LTP, concentrating on the Schaffer collateral‐commissural (SCC) synapse in area CA1 of the hippocampus. These were extremely exciting times for me and led to the accepted mechanism for the induction of LTP via activation of the NMDA receptor, most notably documented in a review that I co‐authored with Tim Bliss in 1993 (Bliss and Collingridge 1993) and summarized in Figure 2.

It was during this time there was an explosion of interest in NMDARs and synaptic function. Most notably, Richard Morris showed that antagonism of NMDARs in the hippocampus resulted in both inhibition of LTP induction in vivo and impairments in spatial learning and memory, as described in another contribution to this collection (Morris 2025). Since this pioneering study, the evidence that NMDARs and LTP underlie many forms of learning and memory is overwhelming.

I have previously written a personal account of these early years, that effectively spans the decade of the 80s, and rather than re‐iterate them here, I refer the interested reader to this publication (Collingridge 2003).

## 4 Birmingham (1990–1994)

In 1990 I was appointed Chair of the Department of Pharmacology at the University of Birmingham in the UK. Here, the lab expanded its interests in two main directions.

### 4.1 Metabotropic Madness

Our investigation of the role of mGluRs in synaptic plasticity was sparked by the development of mGluR ligands, primarily by Jeff Watkins and David Jane (see Box 1 for details of the full chemical names and underpinning pharmacology).

Early on in our investigations, Zuner Bortolotto discovered that a brief application of (1*S*,3*R*)‐ACPD could induce LTP (Bortolotto and Collingridge 1993). The LTP developed slowly over a period of minutes, and fully occluded NMDAR‐LTP and *vice versa*. Interestingly, this (1*S*,3*R*)‐ACPD‐induced slow‐onset potentiation was resistant to antagonism of NMDARs but eliminated by the then new mGluR antagonist MCPG. We concluded that activation of mGluRs could trigger NMDAR‐LTP by bypassing the need for the activation of NMDARs. This begged the question as to what effect the antagonism of mGluRs would have on synaptically‐induced LTP at these synapses. In a project led by Zafar Bashir, we were extremely excited to find that MCPG reversibly blocked the induction of NMDAR‐dependent LTP at SCC synapses without impacting short‐term potentiation (STP) (Bashir, Bortolotto, et al. 1993). Thus, activation of mGluRs could induce, and their antagonism abolish, the induction of LTP. In this study, we also found that MCPG blocked the induction of mossy fiber LTP; a topic to which I will return.

We went on to show that the ability of MCPG to block the induction of LTP depended upon the prior history of synaptic plasticity; prior LTP prevented MCPG from having any effect on subsequent LTP (Bortolotto et al. 1994). We showed that the “priming” was due to activation of mGluRs—since we could prevent the priming with MCPG or induce the priming with (1*S*,3*R*)‐ACPD. In other words, the priming event was remembered during subsequent LTP induction in the same pathway—a phenomenon we termed the “molecular switch”. Cliff Abraham built upon these observations in an elegant set of experiments and named the mGluR‐priming phenomenon, *metaplasticity* (Abraham and Bear 1996).

Our results were rapidly confirmed by some labs and disputed by others. The explanation for this resides in the fact that LTP comprises two mechanistically distinct, but overlapping, processes (termed LTP1 and LTP2) that have differing requirements for mGluR activation—as described in a recent review article that I co‐authored with Cliff Abraham (Collingridge and Abraham 2022).

We were interested in identifying the signaling mechanisms engaged by mGluRs during LTP. We had recently found that conventional LTP, induced synaptically, may require Ca^2+^ release from intracellular stores (Harvey and Collingridge 1992) and identified a similar requirement for LTP induced by (1*S*,3*R*)‐ACPD (Bortolotto and Collingridge 1993).

During this period, we also identified an additional effect of mGluR activation. Jenni Harvey found that a brief application of (1*S*,3*R*)‐APCD caused a rapid increase in the response to the application of NMDA, an effect that was also rapidly reversible upon washout (Harvey and Collingridge 1993). This effect, unlike (1*S*,3*R*)‐APCD‐induced slow‐onset potentiation did not require Ca^2+^ release from intracellular stores. It also did not require PKA or PKC. Interestingly, however, activation of PKC inhibited the effect.

Around this time, long‐term depression (LTD), both *de novo* (Dudek and Bear 1992) and following LTP—depotentiation (Fujii et al. 1991), was shown to depend on the activation of NMDARs. We asked the question of whether mGluRs could also play a role in LTD and found that MCPG could block depotentiation (Bashir, Jane, et al. 1993).

This was also the time when the first mGluR KOs were made. We collaborated with Francois Conquet, who made a KO of mGluR1, and found that, in the absence of this subunit, LTP could still be readily induced in the CA1 and dentate regions of the hippocampus (Conquet et al. 1994). There was, however, a loss of LTP at mossy fiber synapses in area CA3 of the hippocampus and a loss of LTD in parallel fiber to Purkinje cell synapses in the cerebellum.

### 4.2 Calcium Challenges

Based on our observation that NMDARs are required for LTP at CA1 synapses and Gary Lynch's pioneering discovery that the chelation of postsynaptic Ca^2+^ in CA1 neurons blocked LTP (Lynch et al. 1983), it was widely assumed that NMDARs trigger LTP by providing a Ca^2+^ source, a hypothesis strengthened by the finding that NMDARs have a relatively high permeability to Ca^2+^; see, for example, (MacDermott et al. 1986). We set out to test this hypothesis. Simon Alford, a postdoc in the lab at that time, suggested using patch electrodes to load a Ca^2+^ indicator into a single neuron, use the electrode to record synaptic currents and combine this with the then new technique of confocal microscopy. He was the first to achieve this feat and, together with Bruno Frenguelli, demonstrated that synaptic activity drove Ca^2+^ into individual dendritic spines via activation of NMDARs. They also found that the dendritic signal, that was the integration of several spines, was magnified by Ca^2+^ release from intracellular Ca^2+^ stores (Alford et al. 1993) via a mechanism that also involved synaptic activation of mGluRs (Frenguelli et al. 1993).

Achieved in the early 1990s (Collingridge and Abbott 1991), it took several attempts to publish our Ca^2+^ imaging work, for what I believe were not objective scientific reasons—the scars left by this experience remain to this day. I was, however, delighted to see that Tim Bliss, together with Alan Fine and Nigel Emptage, developed this technique further to the extent that they could perform an optical quantal analysis of LTP and provide the strongest evidence to date that LTP involves both pre‐ and post‐synaptic expression mechanisms (Emptage et al. 2003). The combination of whole‐cell or intracellular recording with spine imaging is commonplace today and much simpler to perform given the great advances in imaging technologies.

**FIGURE 3:**
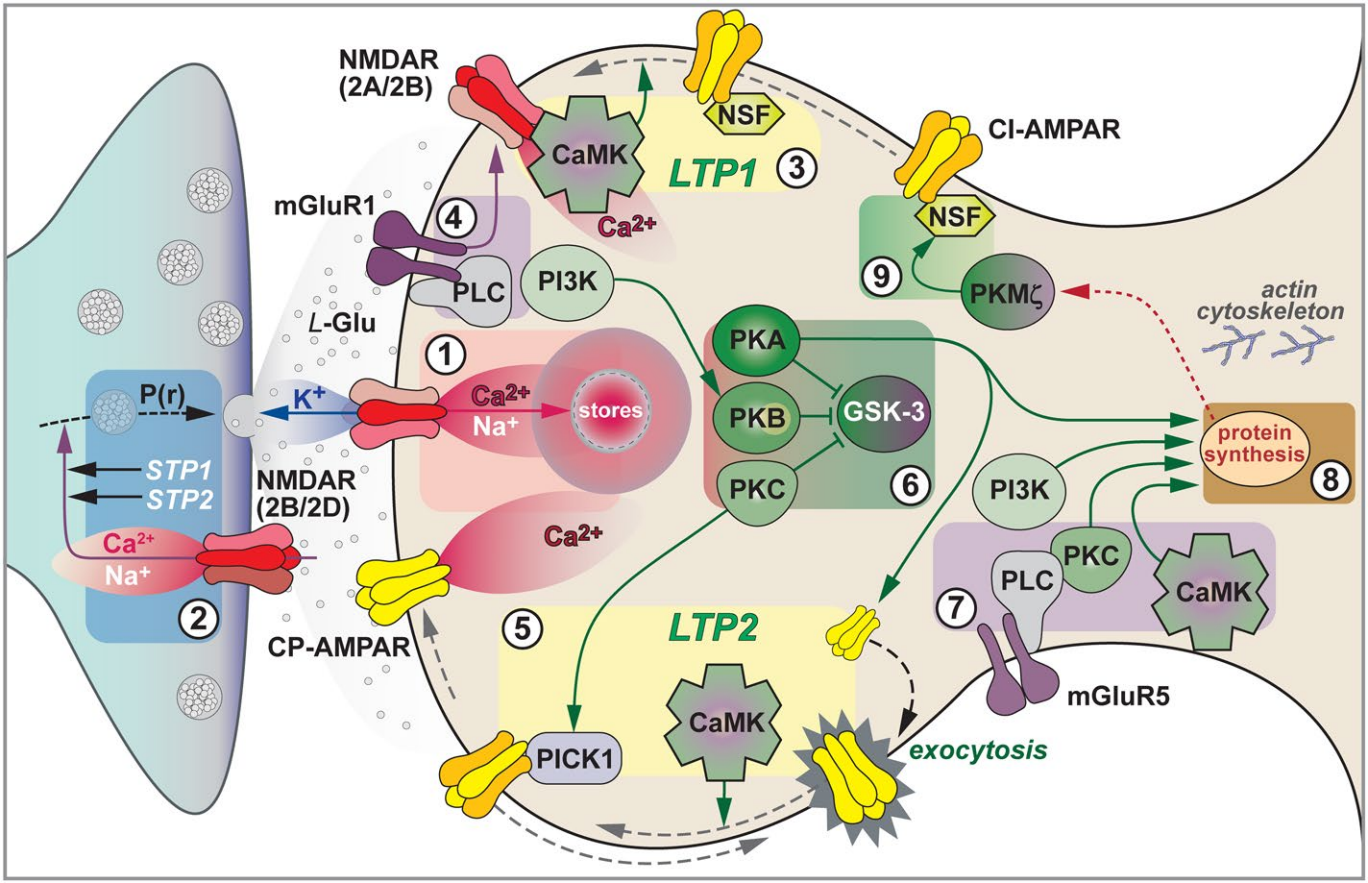
NMDAR‐LTP at SCC synapses (a more contemporary view). 1. Induction. LTP is triggered by the synaptic activation of NMDARs. In adult CA1 these are predominantly GluN2A/2B heterotrimers and their activation results in a net inward current of predominantly Na^+^ (which provides a substantial depolarisation) and ~10% Ca^2+^ (which triggers LTP). The Ca^2+^ signal is greatly magnified by Ca^2+^‐induced Ca^2+^ release. 2. Short‐Term Potentiation. STP is a rapidly developing potentiation that is largely expressed presynaptically, via an increase in P(r). It is induced by high frequency stimulation and decays in a strictly activity‐dependent manner. Two forms of STP can be distinguished: STP1 decays the more rapidly and probably is induced postsynaptically via a GluN2A/2B heterotrimer (with K^+^ potentially serving as a retrograde messenger). STP2 is probably induced via activation of a presynaptic GluN2B/2D heterotrimer. 3. Long‐Term Potentiation: LTP1. The synaptic release of L‐glutamate activates NMDARs and these provide a highly localized increase in cytosolic Ca^2+^ in the postsynaptic neuron. This alone may be sufficient to activate CaMKII, which drives more CI‐AMPARs into the synapse. 4. Co‐induction trigger. Co‐activation of mGluRs (shown here as mGluR1 homodimers) can facilitate the activation of NMDARs, such that they modulate but are not essential for the induction of LTP. 5. Long‐Term Potentiation: LTP2. The Ca^2+^ entering via NMDARs also activates adenylyl cyclase (AC) to generate cAMP and stimulate PKA. This phosphorylates Ser845 of GluA1 to drive CP‐AMPARs onto the plasma membrane but outside of the synapse. A subsequent induction stimulus (within the order of minutes) activates CaMKII to phosphorylate GluA1 on Ser831 to drive the CP‐AMPARs into the synapse in exchange for CI‐AMPARs, via a PICK1‐dependent mechanism. This homo‐exchange provides AMPARs with a higher synaptic single channel conductance, which accounts for LTP2 initially. 6. LTP inhibits LTD. PKB (Akt) is also activated and this inhibits GSK3 to put a brake on LTD. PKA and PKC could serve a similar function too. 7. Metaplasticity. Co‐activation of mGluR5 is also required for induction of LTP2, via a mechanism that involves CaMKII and PKC. The activation of mGlu5Rs can coincide with that of NMDARs or may precede the activation of NMDARs, where it serves as a priming mechanism (a form of metaplasticity). One possibility for a point of convergence of the two induction arms is that the Ca^2+^ entry via CP‐AMPARs fills stores such that mGlu5Rs can trigger Ca^2+^‐induced Ca^2+^ release, enabling the spread of Ca^2+^ within the spine. 8. Protein Synthesis. Low frequency stimulation drives Ca^2+^ through these CP‐AMPARs, a necessary step to trigger *de novo* protein synthesis. This provides a local source of proteins that are required to expand the synapse (and potentially support spinogenesis) and incorporate an increased number of CI‐AMPARs, which underlie the persistent increase in synaptic strength. 9. LTP Maintenance. One such newly synthesized protein is PKMζ which may stabilize newly inserted AMPARs via promotion of the GluA2:NSF interaction. (Note that many molecules are not illustrated, including cytoskeletal components. See text for references).

Much of our studies up to 1993 are summarized in two figures adapted from the review article that I co‐authored with Tim Bliss (Bliss and Collingridge 1993; Figure 2). A more contemporary view of LTP at SCC synapses that incorporates our more recent findings (discussed below) is shown in Figure 3.

## 5 Bristol (1994–2020)

I returned to Bristol in 1994 for a pure research position in the Department of Anatomy. But soon after my arrival, the Head of Department left Bristol and I found myself with another administrative position as Chair of Anatomy. But fortunately I was able to recruit some outstanding independent investigators—Jeremy Henley and Zafar Bashir, soon to be joined by Elek Molnar. In 1999, I stood down from the Chair of Anatomy to become the founding director of the MRC Centre for Synaptic Plasticity. The Centre was a highly multidisciplinary and collaborative environment externally supported mainly by response mode funding from the MRC plus a relatively small infrastructure Centre grant. It had four research pillars: Medicinal chemistry led by David Jane; Molecular sciences led by Jeremey Henley, Cellular sciences led by myself and Systems sciences led by Malcolm Brown. It comprised ~20 PIs and, I believe, was hugely impactful providing an outstanding return on the MRC's modest investment. I remained its director until the MRC decided that MRC Centres should have a finite lifespan and terminated the Centre's funding after 14 years.

### 5.1 AMPAR Wanderings

Our early work had suggested that STP was presynaptic and LTP was, at least in part, postsynaptic—based on iontophoretic sensitivity experiments (Davies et al. 1989). But the underlying mechanisms could only be speculated upon. Fortunately, Tim Benke rejoined my lab as a postdoc and suggested that we could use peak scaled non‐stationary fluctuation analysis to investigate the mechanistic basis of the enhanced AMPAR function in LTP. Together with Andreas Luthi and John Isaac, he showed that LTP was associated with an increase in single channel conductance in some neurons and what was best interpreted as an increase in receptor number in others (Benke et al. 1998). The existence of two mechanisms was perplexing but was finally resolved to my total satisfaction many years later (see below). This triumvirate showed that the increase in single channel conductance was readily reversible by an LTD stimulus (Luthi et al. 2004). These initial experiments were done in young rats of ~2 weeks of age. We also examined rats of ~1 week of age and found further complexity in the expression mechanisms. In work led by Mary Palmer (Palmer et al. 2004), we found that some neurons exhibited pronounced increases in probability of release—P(r)—and others displayed a *reduction* in single channel conductance. One scenario to explain these results is as follows: Initially synapses have a low P(r). In response to the initial LTP stimulus, they increase their P(r). These young synapses operate, at least in part, via activation of calcium permeable (CP) AMPARs. The second LTP stimulus replaces CP‐AMPARs with a greater number of lower conductance calcium impermeable (CI) AMPARs. Thereafter, an additional LTP stimulus may either drive more CI‐AMPARs into the synapse or lead to the insertion of new CP‐AMPARs, potentially as a 1:1 exchange with CI‐AMPARs.

To provide more direct evidence for or against alterations in receptor number we used two complementary approaches. Elek Molnar developed the first glutamate receptor antibodies (AMPAR and NMDAR) for live cell labelling (Richmond et al. 1996; Pickard et al. 2000) and Jeremy Henley was the first to tag glutamate receptors with GFP (Doherty, Coutinho, et al. 1999) and pHlourins (Ashby et al. 2004) for dynamic studies of recombinant receptor trafficking. Using these and related tools, several other labs, particularly that of Robert Malinow, provided evidence for changes in AMPAR number during LTP and LTD (reviewed in Collingridge et al. 2004).

Our contributions to this emerging field included visualization of glutamate receptors. Using Molnar's antibodies we found evidence for a developmental regulation in the surface expression of AMPARs and NMDARs (Pickard et al. 2000), consistent with the electrophysiological studies. We also found that during a form of chemical LTP, induced by very brief depolarization (Fitzjohn, Pickard, et al. 2001), there was an increase in the surface expression of AMPARs, measured using a pan‐AMPAR antibody (Pickard et al. 2001). Using pHluorin‐GluA2, in a study led by Mike Ashby, we found that, in response to a challenge with NMDA, AMPARs internalized from extrasynaptic sites in advance of synaptic loss (Ashby et al. 2004). This finding was consistent with the emerging view that LTD involves a two‐step process comprising transport/diffusion out of the synapse followed by endocytosis. Imaging techniques have, of course, improved greatly since these early days—and important details have been added to the trafficking process that underlies many aspects of synaptic plasticity.

To gain insights into the molecular basis of AMPAR trafficking, Jeremy Henley, initially with Shigetada Nakanishi, looked for direct binding partners and demonstrated that N‐ethylmaleimide sensitive factor (NSF) binds to the GluA2 subunit (Nishimune et al. 1998) (as did Rick Huganir and Ed Ziff at around the same time). John Isaac explored the functional consequences of disrupting the GluA2:NSF interaction using interfering peptides. We had predicted that NSF would be important for LTP but found, to our surprise, that disruption of the GluA2‐NSF interaction led to a rapid rundown in basal synaptic transmission (Nishimune et al. 1998). This showed that AMPARs are mobile and stabilized at the synapse via their interaction with NSF. Jaques Noel went on to show that disrupting the NSF‐GluA2 interaction resulted in fewer AMPARs on the neuronal surface (Noel et al. 1999). Subsequently, Andreas Luthi found that the AMPARs stabilized by the NSF interaction were the population that were removed from the synapse during LTD (Luthi et al. 1999). Some years later, Todd Sacktor, who discovered the critical role of PKMζ in the maintenance of LTP (Shema et al. 2007), showed that the mechanism of increased AMPAR expression involves a stabilization of the NSF:GluA2 interaction (Yao et al. 2008).

An important breakthrough, by Yu Tian Wang and Morgan Sheng, was the finding that AP2 competes with a similar binding site as NSF on the c‐terminal tail of GluA2 (Lee et al. 2002). Work led by Jeremy Henley went on to show that hippocalcin, a member of the high affinity neuronal calcium sensor (NCS) family, was important for LTD. It was proposed that on sensing Ca^2+^, hippocalcin targeted AP2 to the plasma membrane to enable its exchange for NSF on GluA2 and initiate clathrin‐mediated endocytosis (Palmer et al. 2005).

We also studied PICK1, another GluA2 interacting partner identified independently by Henley and Huganir, and, in work led by John Isaac, found that it regulates the GluA2 content of AMPARs at the synapse (Terashima et al. 2004) and is important for bi‐directional synaptic plasticity (Terashima et al. 2008). Jon Hanley, another MRC Centre PI, “picked up” on PICK1 and has provided major insights into its functions in synaptic plasticity (Hanley 2018). These studies, initially with NSF, heralded in a large international effort to decipher the molecular basis of AMPAR trafficking and its role in synaptic plasticity, as described in more detail in (Collingridge et al. 2004) and (Bliss et al. 2024).

Zhengping Jia (Toronto), in the laboratory of John Roder, generated mice lacking the GluA2 subunit and hence are calcium permeable (CP). He made the pivotal discovery that CP‐AMPARs could mediate a form of LTP that was independent of and additive to classic NMDAR‐dependent LTP (Jia et al. 1996). In Bristol, John Isaac extended this work by showing that CP‐AMPARs could be necessary for the induction of LTP in wildtype rodent hippocampus (Plant et al. 2006). Another controversy followed but is now readily reconcilable, as discussed later in the article.

### 5.2 NMDAR Nuances

Since our early studies, NMDARs have been cloned, multiple subunits identified and KOs of every subunit made. Many labs addressed the subunit compositions of the NMDARs that mediate LTP and LTD. Most impactful, Yu Tian Wang (Liu et al. 2004) and Zafar Bashir (Massey et al. 2004) showed that LTP involved GluN2A and LTD involved GluN2B subunits in the hippocampus and perirhinal cortex, respectively. In our hippocampal studies, based on a detailed pharmacological characterization of antagonists (Box 1), we concluded that the principal subunit that underlies LTP at SCC synapses is a GluN2A/GluN2B heterotrimer (Bartlett et al. 2007; Volianskis et al. 2013). With respect to LTD our data supported the role of a GluN2B heterodimer under some (France et al. 2017) but not all (Bartlett et al. 2007) conditions. It seems likely that a GluN2B/2D heterotrimer can mediate LTD under some circumstances.

An additional level of complexity was discovered when we examined STP. Prior to joining the laboratory, Arturas Volianskis, working in the lab of Morten Jensen (Aarhus), had identified some remarkable properties of STP (see (Volianskis et al. 2015)), most notably that its decay is strictly activity‐dependent. In Bristol, he found that STP comprises two kinetically distinct components with the fast component (termed STP1) involving GluN2A and GluN2B subunits and the slower component (STP2) involving GluN2B and GluN2D subunits (Volianskis et al. 2013). Interestingly, STP2 is exquisitely sensitive to blockade by ketamine (Ingram et al. 2018). Ketamine is a non‐competitive NMDAR antagonist, as first shown by David Lodge (Anis et al. 1983), and possesses rapid and relatively long‐lasting antidepressant activity. It is conceivable that an effect on STP explains some of its antidepressant actions.

### 5.3 KAR Complexities

The development of pharmacological tools that effectively blocked KARs (see Box 1) enabled their functions to be established. We identified several of these.

**FIGURE 4:**
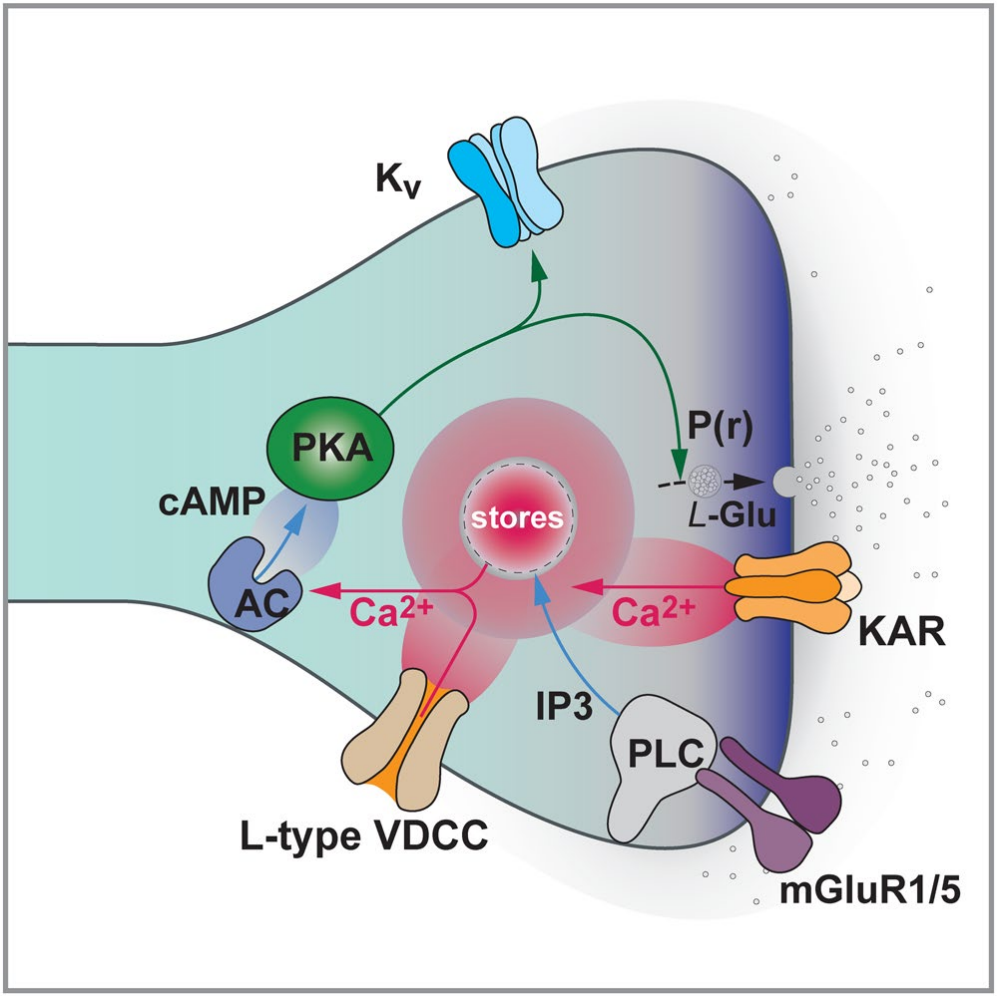
Mossy fiber LTP. Prior work had provided strong evidence that mossy fiber LTP was expressed by an increase in the probability of L‐glutamate release and involved a PKA‐dependent mechanism. We added roles for mGluRs, KARs, and Ca^2+^ stores in the process. We propose that synaptically released L‐glutamate activates presynaptic mGluRs and KARs. At the time that these experiments were conducted, we assumed that both mGluR1 and mGluR5 were activated but that either was sufficient. (This was because inhibition of both mGlu1 and mGlu5 was required to block LTP but activation of only mGlu1 or mGlu5 was sufficient to induce LTP—when applied with a GluK1 agonist). In light of more recent work, it is far more likely that a mGlu1/mGlu5 heterodimer (which has this exact pharmacology) is responsible. We also proposed that a GluK1‐containing KAR was necessary and that its role is to provide a Ca^2+^ signal (analogous to NMDARs at CA1 synapses). Hypothetically, mGluRs provide IP3 and KARs provide Ca^2+^, which act synergistically to release Ca^2+^ from stores. This could enable the spread of Ca^2+^ to act on a Ca^2+^‐sensitive adenylyl cyclase to trigger the cAMP/PKA cascade. Ca^2+^ entry via L‐type Ca^2+^ channels may also participate. PKA may result in a persistent increase in basal Ca^2+^ due to regulation of K^+^ channels. Figure adapted from (Nistico et al. 2011).

1. Initially, we worked with Jeremy Henley to identify a presynaptic kainate receptor at excitatory synapses (Chittajallu et al. 1996)
2. Next, in collaboration with David Lodge at Eli Lilly, Vernon Clarke identified a role of kainate receptors regulating GABAergic inhibition (Clarke et al. 1997).
3. Studies conducted by Michel Vignes identified a role of KARs in synaptic transmission at the mossy fiber pathway in the hippocampus (Vignes and Collingridge 1997).
4. Thereafter, studies led by Zuner Bortolotto identified a role for kainate receptors in mossy fiber LTP (Bortolotto et al. 1999). See Figure 4.
5. Work led by Sari Lauri identified roles of KARs in frequency facilitation at mossy fiber synapses (Lauri et al. 2001).
6. She also identified a role of Ca^2+^ release from intracellular stores in this process (Lauri et al. 2003).
7. At CA1 synapses, she found that KARs were important early in development, where they provided a tonic regulation of glutamate release (Lauri et al. 2006). Interestingly, this early form of LTP involves a down‐regulation of these presynaptic KARs resulting in a persistent increase in P(r) (Lauri et al. 2007).
8. Ilse Smolders found that KAR antagonists reduce limbic seizure (Smolders et al. 2002).
9. In a collaboration with the late Bob Muller, John Huxter showed that KARs regulate the frequency of theta oscillations (Huxter et al. 2007).
10. Vernon Clarke showed that KARs expressed on interneurons in area CA1 contributed to the regulation of the induction of LTP (Clarke et al. 2012).

Our work was, of course, not done in a vacuum: the availability of selective compounds together with the KOs generated in the Steve Heinemann lab led to several similar studies. Key labs in this field included those led by Nicoll, Lerma and Mulle. See (Jane et al. 2009) for a more detailed discussion of our work placed in the context of the work of other groups and a discussion of the subtype selectivity of the ligands used. Unsurprisingly, there were many agreements but also some disagreements, in particular with respect to the role of KARs in LTP and frequency facilitation at mossy fibers.

In this respect, some labs agreed that KARs were important but disagreed on the subtype involved while others concluded that mossy fiber LTP and frequency potentiation was completely independent of KARs. We had suggested that the KAR contained a GluK1 subtype since, despite the relatively low expression of this subtype at mossy fibers, we could block the induction of mossy fiber LTP with six different GluK1 antagonists (Jane et al. 2009). To further address the controversy we performed two complementary sets of experiments.

1. We compared two different slice orientations—the more commonly used transverse and our standard parasagittal slices. We confirmed that in transverse slices GluK1 antagonists were ineffective. Thus the GluK1 sensitivity of mossy fiber LTP and frequency facilitation was specific to the latter orientation, potentially because this better preserves the mossy fiber input onto CA3 pyramidal neurons (Sherwood et al. 2012).
2. To remove ambiguity of the pathway under investigation, we used a method developed by Dmitri Rusakov (Scott and Rusakov 2006). This involved using a patch electrode to record from a granule cell, to load the axon with a Ca^2+^ indicator and to deliver a brief train of action potentials. The sensitivity of the Ca^2+^ signals in visually‐identified single giant mossy fiber boutons to KAR antagonists was then assessed. Sheila Dargan demonstrated that the facilitation of the Ca^2+^ signal was blocked by the potent and selective KAR antagonist ACET (Dargan et al. 2009). These observations strongly suggest that mossy fiber boutons express KARs (Nistico et al. 2011).

In my opinion, the simplest explanation for these observations is that LTP and frequency facilitation at giant mossy fiber synapses are both induced by a presynaptic GluK1‐containing KAR.

### 5.4 More Metabotropic Madness

#### 5.4.1 Mossy Fiber LTP

As mentioned above, we had shown that mossy fiber LTP was sensitive to both antagonists of mGluRs and KARs. To explore their relationship we used two approaches (Nistico et al. 2011). Using field potential recording, Robert Nistico discovered that the combination of 3,5‐dihydroxyphenylglycine (DHPG), which selectively activates the group I mGluRs (mGluR1 and mGluR5), and ATPA, a GluK1‐selective agonist, induced a slowly developing potentiation at mossy fiber synapses. (The pharmacology of the DHPG action is consistent with a mGlu1/mGlu5 heterodimer working in concert with a GluK1‐containing KAR). Like synaptically‐induced mossy fiber LTP, the ATPA+DHPG potentiation depended upon activation of PKA and Ca^2+^‐induced Ca^2+^ release. Using the Ca^2+^ imaging approach, described above, Sheila Dargan and Mascia Amici found that frequency facilitation at single mossy fiber boutons was prevented by inhibition of either group I mGluRs or KARs and that co‐application of ATPA and DHPG resulted in a facilitation of the Ca^2+^ signal and an increase in basal bouton Ca^2+^, via a mechanism that involves Ca^2+^‐induced Ca^2+^ release. Based on these findings we proposed a mechanism for the induction of mossy fiber LTP (Figure 4).

Given the differences in KAR involvement in mossy fiber LTP according to slice orientation, we wished to know how KARs would contribute to LTP in vivo. In pentobarbitone‐anesthetized rats, high frequency stimulation evoked an NMDAR‐independent slow onset potentiation (Wallis et al. 2015), as described previously (Derrick and Martinez Jr. 1994). Unlike the situation in slices, James Wallis was unable to block mossy fiber LTP with either mGluR antagonists or KAR antagonists applied alone. However, the combined application of mGluR1, mGluR5, and GluK1 KAR antagonists fully blocked the induction of mossy fiber LTP. These findings confirm, in vivo, that both group I mGluRs and GluK1‐containing KARs are important for mossy fiber LTP. The reason that activation of either mGluRs or KARs is sufficient in vivo (hence blocking both receptor types is necessary) while activation of both is required in vitro (such that blocking one receptor type is effective) is not known. We speculated, however, that it may relate to differences in the state of filling of internal Ca^2+^ stores.

#### 5.4.2 CA1 LTP

We also built upon our initial studies on the roles of mGluRs in LTP, LTD and metaplasticity. We used the mGluR1 KO provided by Conquet, the mGluR5 KO provided by Zhengping Jia and well‐characterized pharmacological tools, such as MPEP. We found that LTP could still be induced in area CA1 with both group I mGluRs completely knocked out. However, we discovered that activation of mGluR5 was absolutely required for the priming of synaptic plasticity (Bortolotto et al. 2005). In 2007, Zhengping Jia joined the laboratory for a sabbatical and we continue to explore the functions of mGlu5 receptors. For example, we varied the stimulus parameters used for priming and found that just a few stimuli (between 5 and 8) were necessary to fully engage this form of metaplasticity (Bortolotto et al. 2008). Prior to this, it was widely assumed that mGluRs require prolonged synaptic activation. I consider this a significant observation, but was one that has flown under the radar. In terms of the signaling mechanisms involved in priming, we found evidence for roles of CaMKII (Bortolotto and Collingridge 1998) and PKC (conventional isoforms) but not PKA (Bortolotto and Collingridge 2000). Note that CaMKII has been extensively studied in the context of synaptic plasticity and has several roles beyond metaplasticity, including structural roles (see Nicoll 2025).

We also extended our studies of slow‐onset potentiation induced by (1*S*,3*R*)‐ACPD. We found that this form of LTP requires Ca^2+^ signaling in the postsynaptic neuron and is associated with a postsynaptic increase in AMPAR sensitivity (Bortolotto and Collingridge 1995). However, unlike conventional LTP, there is a requirement for activity to be transmitted from area CA3 (Bortolotto and Collingridge 1995). This is likely due to the need for activity to load the intracellular Ca^2+^ stores, which are typically depleted at rest; this can be achieved either by Ca^2+^ entry via NMDARs or L‐type Ca^2+^ channels (Rae et al. 2000).

#### 5.4.3 CA1 LTD

**FIGURE 5:**
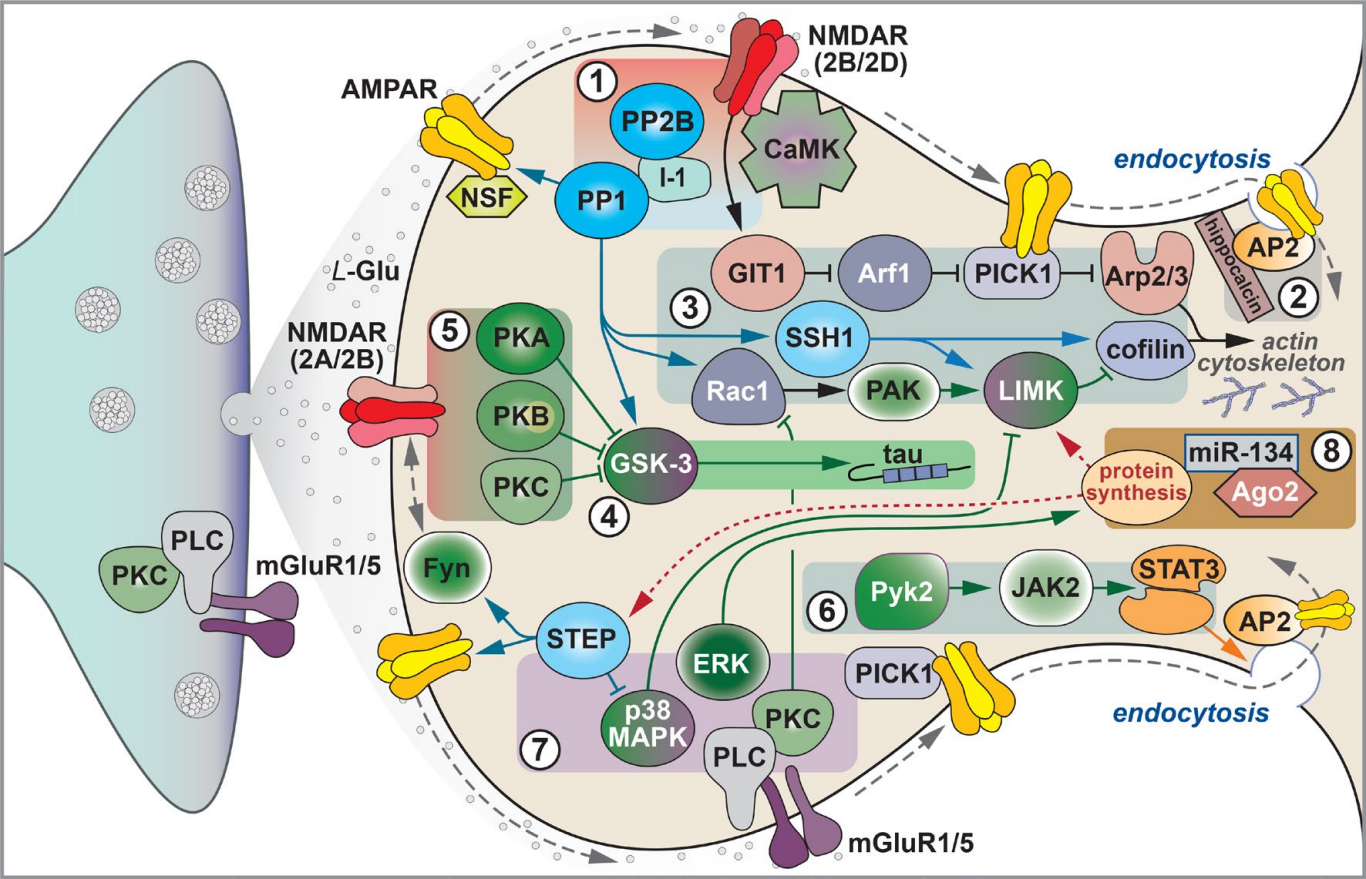
NMDAR‐LTD at SCC synapses. Induction 1. Activation of NMDARs can induce LTD. It is assumed that certain patterns of activity (e.g., prolonged 1–5 Hz stimulation) trigger receptors located outside of the postsynaptic density. These likely comprise GluN2B heterodimers or GluN2B/D heterotrimers. Ca^2+^ influx activates a canonical Ser/Thr protein phosphatase (PP) cascade, one function of which may be to dephosphorylate the c‐terminal tail of GluA1. This may enable lateral diffusion of AMPARs out of the synapse. 2. Endocytosis. AMPARs are stabilized at the plasma membrane by a direct interaction with NSF. On sensing Ca^2+^, hippocalcin, a member of the high affinity neuronal calcium sensor (NCS) family, brings AP2 to the GluA2 c‐terminal tail and initiates its exchange with NSF to enable clathrin‐mediated endocytosis. 3. Cytoskeleton. PP1 also helps trigger a cascade that activates cofilin to lead to actin depolymerisation. A key node in this cascade is LIMK, which is immediately upstream of cofilin and is regulated by miR‐134 and Argonaute‐2. The Arp2/3 complex is also activated to enable actin branching. Central to this cascade is PICK1, which links endocytosis and actin changes. 4. Regulation. GSK3 is a master regulator of LTD, integrating many signals and controlling numerous effector mechanisms. Here is illustrated the direct phosphorylation of tau, which most probably orchestrates actin/microtubule re‐arrangements. 5. Significantly, GSK3 is inhibited during LTP via PKB (Akt) and, most probably, other kinases that directly phosphorylate the enzymes (at Ser 21 in GSK3α and Ser 9 in GSK3β). This limits the LTD process, enabling enhanced LTP. 6. Tyrosine phosphorylation. JAK2/STAT3 is also involved in LTD, where it might coordinate cytokine signaling and synaptic plasticity. Pyk2 is a potential upstream regulator. 7. Metabotropic Glutamate Receptors. mGluRs also trigger LTD that involves trafficking of AMPARs. This engages a different signaling cascade (e.g., a protein kinase, p38MAPK and a protein tyrosine phosphatase, STEP). mGluR‐LTD can also be expressed presynaptically, via an increase in P(r). (Only a subset of the known signaling molecules involved in LTD are illustrated. See text for more details and references).

We also extended our studies on LTD (Figure 5). Notably, we examined the effect of the group I mGluR agonist, DHPG, and found that it induces a form of LTD (Palmer et al. 1997; Fitzjohn et al. 1999). DHPG‐LTD is a very robust phenomenon, which has since been used in many hundreds of publications. We mainly used DHPG to investigate the signaling mechanisms involved in mGluR‐triggered LTD. Somewhat to our surprise, the signaling involved in DHPG‐LTD is very different from that involved in mGluR priming, (1*S*,3*R*)‐ACPD‐induced slow onset potentiation and DHPG‐induced facilitation of NMDAR responses. DHPG‐LTD was insensitive to depletion of intracellular Ca^2+^ stores, inhibition of PKA and PKC and was actually potentiated by inhibition of CaMKII. In contrast to NMDAR‐LTD, which requires activation of a Ser/Thr phosphatase cascade (Mulkey et al. 1993), we observed facilitation of DHPG‐LTD when we inhibited PP1/PP2A and no effect of inhibition of PP2B (Schnabel et al. 2001). Instead, we discovered that DHPG‐LTD requires activation of a protein tyrosine phosphatase (PTP) (Moult et al. 2002, 2006). Furthermore, we found that co‐activation of PTPs and p38MAPK is sufficient to induce mGluR‐LTD (Moult et al. 2008). Subsequently, in collbaoration with Paul Lombroso (Yale), we identified striatal‐enriched protein phosphatase (STEP) as a critical component for mGluR‐LTD (Zhang et al. 2008). It is noteworthy that we have never observed a dependence of mGluR‐LTD on *de novo* protein synthesis (Moult et al. 2008), which contrasts with some other studies (Huber et al. 2000). Our work with STEP offers a potential explanation. STEP is rapidly synthesized in response to mGluR activation (Zhang et al. 2008). Potentially, there is sufficient STEP to sustain mGluR‐LTD ordinarily but with time, perhaps as slices deteriorate, new STEP is needed to be synthesized.

It should come as no surprise that mGluR signaling is complex, given that mGluRs are G‐protein coupled receptors that initiate diverse signaling cascades. But it did make the identification of their roles in synaptic plasticity challenging and sometimes controversial. In conclusion of this section, our work had shown that the activation of NMDARs alone may be sufficient to induce LTP (i.e., LTP1) or may require co‐activation of mGluRs (i.e., LTP2), depending on the induction protocols and impact of metaplastic events. The extent to which these complexities, that provide huge modulatory potential, apply to other pathways in the CNS is largely unexplored.

### 5.5 Alzheimer's Disease

Around the turn of the millennium, we were approached by Guy Seabrook (MSD) to study LTP in mouse models relevant to AD. The first of these was a mouse in which amyloid precursor protein (APP) had been knocked out. We observed a deficit in LTP (Seabrook et al. 1999) that may be due to alterations in synaptic inhibition and depends on GABA‐B regulation (Fitzjohn et al. 2000). Second, we studied a presenilin‐1 (PS1) heterozygous KO and observed a substantial LTP deficit when the induction trains were spaced apart (Morton et al. 2002). Both of these findings suggest that APP and PS1 have normal synaptic functions and, consequently, mutations in these early‐onset AD genes may, in part, contribute to neurodegeneration as a consequence of their loss of function.

Next we studied mice that overexpressed the Swedish mutant APP (K670N and M671L) (Fitzjohn, Morton, et al. 2001) or both this mutant form of APP plus a PS1 mutation (A246E) (Fitzjohn et al. 2010). We aged the mice for up to 18 or 14 months, respectively. To our surprise, we did not observe any LTP deficits, though we did detect an age‐dependent reduction in synaptic transmission. A similar conclusion had been reached by Roger Nicoll (Hsia et al. 1999). Since these early days, many studies have supported this conclusion while others have reported impairment in LTP, as we discussed previously (Nistico et al. 2012). (Note that LTP deficits may result from alterations in the LTP process or an indirect consequence of altered synaptic transmission due to the property of cooperativity).

### 5.6 Phosphorylation Perplexities

An obvious question that numerous labs have addressed is what leads from the synaptic activation of NMDARs to the increase in AMPAR function and increase in P(r) that underlie LTP at SCC synapses. Our review in 1993 simply stated “kinases”. Over the ensuing years, several kinases have been shown to play a role in LTP and several phosphatases in LTD. For a summary of this extensive literature see (Bliss et al. 2024). Our labs' work initially focused on PKC, where we obtained evidence that conventional isoforms were not involved in LTP1 but rather were involved in its priming (Bortolotto and Collingridge 2000) and PICK1‐dependent re‐potentiation (Daw et al. 2000) at SCC synapses.

Another kinase we studied was PI3K. Shahid Zaman was interested in this kinase and in work led by John Isaac, we made the surprising discovery that PI3K was acting to maintain input specificity of LTD at SCC synapses (Daw et al. 2002). This led to my interest in GSK3, a classical downstream effector of PI3K. To my surprise, when Stephane Peineau inhibited GSK3, NMDAR‐dependent LTD was eliminated (Peineau et al. 2007, 2009). This observation shifted my attention to NMDAR‐LTD (Figure 5).

**FIGURE 6:**
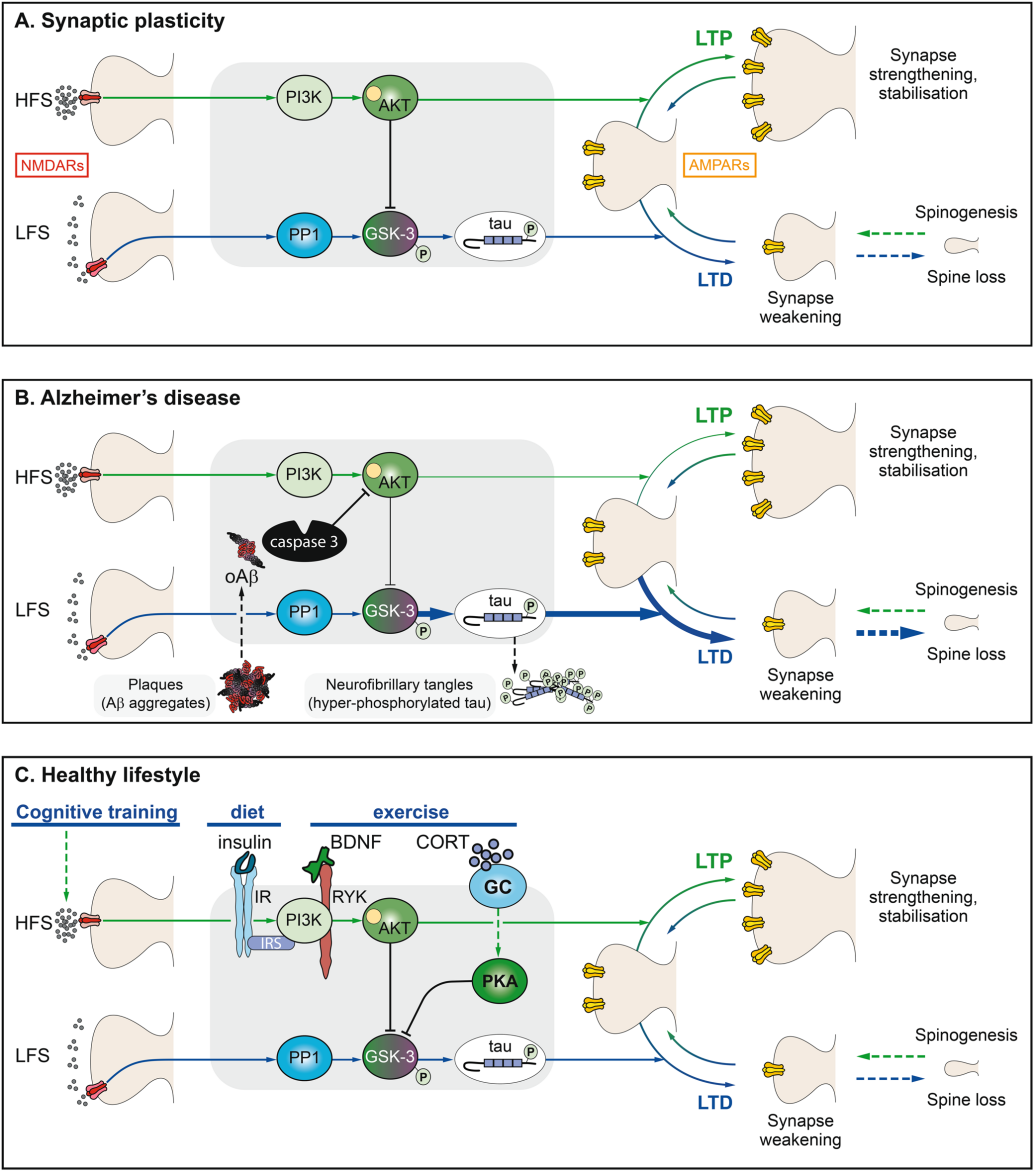
Synaptic Plasticity in Health and Disease. (A) The activation of NMDARs, typically by high frequency stimulation (HFS), leads to long‐term potentiation (LTP), which involves an increase in the number and properties of synaptic AMPARs (Benke et al. 1998). There may also be an increase in spine size and/or number. In contrast, low‐frequency stimulation (LTD) typically induces long‐term depression (LTD) and spine shrinkage. These processes are readily reversible. Amongst the many molecular components that link NMDAR activation to alterations in AMPAR function is GSK3 (Peineau et al. 2007). During LTD, GSK3 is activated by dephosphorylation and it then phosphorylates numerous substrates, including tau (Kimura et al. 2014). During LTP, GSK3 is inhibited by phosphorylation via a pathway involving PI3K and AKT (PKB). Critically, when LTP is induced, LTD is suppressed via this pathway (Peineau et al. 2007). (B) In many brain disorders, the LTP/LTD balance becomes upset, as exemplified here in the context of AD. Once toxic oligomeric species of Abeta (Aβ) get into a synapse (Whitcomb et al. 2015), they can rapidly trigger a cascade that results in caspase‐3‐mediated cleavage of AKT (Jo et al. 2011). This results in run‐away activation of tau leading to excessive LTD and, if left unchecked, irreversible synapse and eventually neuron loss. The hyperphosphorylated tau accumulates as tangles marking the dead neurons. This pathway links plaques to tangles in a well‐defined molecular cascade. (C) Lifestyle affects synaptic health via this pathway. For example, cognitive training most probably engages the LTP cascade, and thereby keeps LTD in check. “A crossword a day keeps the doctor away.” One result of diet is the level of activation of insulin receptors, which is likely protective of synapses via the classical insulin signaling pathway (Insulin receptor (IR)—PI3K—PKB (AKT)—GSK3). Insulin resistance would be expected to reduce this protective effect. Exercise increases BDNF, which also signals via PI3K and may promote LTP and reduce LTD via this pathway. Exercise also increases cortisol/corticosterone, which activates PKA. PKA also phosphorylates GSK3 to inhibit its function. Acute stress enhances LTP via activation of glucocorticoid receptors (GCs), activation of PKA, and insertion of CP‐AMPARs (Whitehead et al. 2013). More generally, many lifestyle factors may shift the LTP/LTD balance via regulation of the core GSK3 pathway.

At this point I took a sabbatical in Vancouver and collaborated with Yu Tian Wang on the role of GSK3 in synaptic plasticity. We found that GSK3 was activated by PP1, which was already an established mediator of NMDAR‐LTD (Mulkey et al. 1993). But most strikingly, we found that GSK3 was inhibited by PI3K via activation of PKB (Akt). Since the PI3K/PKB pathway is activated during LTP, this observation showed how LTP can inhibit LTD directly. Indeed, we directly demonstrated that this was the case. I believe that the significance of this molecular interaction cannot be overstated. Based on this work, and the parallel finding that overexpression of GSK3 inhibits LTP (Hooper et al. 2007), we proposed that GSK3 plays a crucial role in synaptic pruning, particularly early in development, but in adulthood its activity is limited by LTP (Collingridge et al. 2010). However, if this regulation is disrupted, then GSK3 activity increases and aberrant synaptic elimination ensues. Since increased GSK3 activity is correlated with AD, we proposed that breakdown of the LTP inhibition of LTD is a key step in the generation of this, and potentially other, neurodegenerative disease (Collingridge et al. 2010). Furthermore, it seems reasonable to speculate that the PI3K/PKB/GSK3 axis is a key hub whereby lifestyle factors can negatively or positively impact cognitive function. If correct this molecular cross‐talk has huge implications for brain health, as depicted in Figure 6.

GSK3 is a molecular organizer of cellular events, integrating a variety of signals and regulating hundreds of substrates. Since GSK3 hyperphosphorylates tau in AD, we wondered whether tau is also a physiological substrate of GSK3 during LTD. Such a role would be consistent with the hypothesis that AD is caused by a dysregulation of the physiological process of LTD (or more specifically the LTP/LTD balance). In a study led by Kei Cho, we indeed provided evidence that tau is a downstream effector of GSK3 during LTD (Kimura et al. 2014), thus providing very strong support for the LTP/LTD imbalance hypothesis. We also searched for other synaptic substrates of GSK3 and, in collaboration with Adam Cole, identified a role for phosphatidylinositol 4‐kinase type II alpha (PI4KIIα) in the regulation of synaptic NMDARs (Amici et al. 2021). Work from other labs has identified additional synaptic GSK3 substrates, and a complex picture of synaptic orchestration is beginning to emerge (see Bliss et al. 2024).

There is compelling evidence that classical Alzheimer's disease (AD) is triggered by toxic species of Aβ, a cleavage product of amyloid precursor protein (APP). In pioneering studies, Roger Anwyl and Michael Rowan (Trinity College, Dublin) demonstrated that Aβ acutely inhibited LTP (Rowan et al. 2005) and later they showed that it enhanced LTD (Kim et al. 2001). These extensively studied acute actions of Aβ on synaptic plasticity suggest that AD is caused by an acute toxic action of Aβ, the extremely slow development of AD being due to the time it takes for toxic levels to reach synapses. Indeed, the earlier observation described above that LTP is preserved but synaptic transmission impaired in AD mice is consistent with this notion. Thus, at any given instance in time, a synapse may have experienced the acute toxicity (resulting in its loss and hence less synaptic transmission overall) or is still unaffected (so LTP is normal). In this scenario, which we consider extremely likely, the study of the acute synaptic toxicity of Aβ is highly relevant to understanding the mechanistic basis of the chronic disease.

In this respect, in studies again led by Cho, it was shown that the acute ability of Aβ to inhibit LTP was due to activation of GSK3 (Jo et al. 2011). Thus, acute inhibition of GSK3 could reverse the LTP‐inhibiting action of Aβ. Building on an earlier study on LTD mechanisms by Cho and Morgan Sheng, we showed further that the pathway involved caspase‐3 and cleavage of PKB (Jo et al. 2011). In a related study, Ole Paulson (Cambridge, UK) showed that the Aβ‐mediated inhibition of LTP was absent in tau KO mice (Shipton et al. 2011). Considered together, these findings provide a mechanistic link between Aβ and tau in the initiation of synaptic weakening that triggers AD (see Figure 6). In further work, also led by Cho, it was shown that if Aβ is directly loaded into a neuron, via a patch pipette, there is a rapid (within min) PKA‐dependent insertion of CP‐AMPARs (Whitcomb et al. 2015). This could be relevant to the early detrimental effects of Aβ, since prolonged Ca^2+^ entry via CP‐AMPARs is known to be toxic to neurons.

Alterations in microglia have been strongly implicated in AD and shown to contribute to mGluR‐LTD by the Trinity College group (Wang et al. 2004). Building upon this, we found that the facilitation of mGluR‐LTD by Aβ requires activation of complement C5aR1 signaling (Ng et al. 2023). Numerous other studies on the acute synaptic effects of Aβ have revealed many mechanistic insights into the early pathological changes that occur in AD.

Our work with GSK3 refuted the simple notion that bidirectional synaptic plasticity was due to phosphorylation events that were under the control of kinases and phosphatases for LTP and LTD, respectively. Rather, these individual forms of synaptic plasticity can involve the interplay of kinases and phosphatases. We then searched for the involvement of additional kinases in NMDAR‐LTD; we found no evidence for additional Ser/Thr kinases (Peineau et al. 2009) but, in a study led by Celine Nicolas, we unexpectedly identified a role for a Tyr kinase, JAK2 (Nicolas et al. 2012). JAK2 typically signals via phosphorylation of STAT3 to regulate transcription in response to signals such as cytokines. She found that STAT3 was also required for LTD via a cytoplasmic action (Nicolas et al. 2012, 2013), though later effects involving transcription cannot be excluded (and indeed seem likely).

### 5.7 The Cytoskeleton

Central to both LTP and LTD are alterations in the synaptic cytoskeleton; a topic of great importance but one that we have only occasionally delved into. In addition to tau, described earlier, we have provided electrophysiological support for Jon Hanley's work on the role of the actin cytoskeleton in NMDAR‐LTD. In the first study, a pathway was defined that triggered the activation of the Arp2/3 complex, a molecule required for the formation of actin branching (Rocca et al. 2013). Thus, during LTD, NMDAR activation triggers GIT1, which activates Arf1 that binds PICK1 to limit its inhibition of Arp2/3. In the second study, it was found that Argonaute 2, a major component of the RNA‐induced silencing complex, is involved in NMDAR‐LTD (Rajgor et al. 2018). In terms of its action on spine morphology, the mechanism involves a translational suppression of LIMK by miR‐134. It should be noted that LIMK, together with activators (PAKs) and effectors (cofilin), had previously been shown to be critical for synaptic plasticity by Zhengping Jia, who generated KOs for these cytoskeletal components (Meng et al. 2002).

## 6 Seoul (2009–2013)

I joined the World Class University (WCU) program that was initiated in South Korea to encourage international collaboration. I spent one semester per year at Seoul National University (SNU) for 5 years working closely with Bong‐Kiun Kaang, our local host, and Min Zhou, from the University of Toronto. After the position officially ended in 2013, we have maintained a collaboration to this day. Much of our work has centred around the anterior cingulate cortex (ACC) and so will not be described in this hippocampus centric article.

Returning to the hippocampus, I was involved in one of Bong‐Kiun Kaang's projects that identified a role for PI3Kγ in hippocampal LTD (Kim et al. 2011). My Seoul lab was run full‐time by Tom Sanderson. He focused on multiphoton imaging and identified how AMPAR internalization was dependent on P(r), due to a mechanism involving activity‐dependent internalization of mGluR1 (Sanderson et al. 2018). He also supervised two Master's students. One of these, Yeseul Lee, continued the lab's interest in GSK3 and found that inhibition of the kinase enhanced cognition (Lee et al. 2021), suggesting that there is a tonic brake on LTP imposed by the constitutive activity of the enzyme.

The second, Pojeong Park built on the pioneering discovery by Reymann and Matthies that LTP can require de novo protein synthesis. As described in a recent review (Bliss et al. 2018), the Magdeburg team had showed that repeated tetani spaced minutes apart could induce a protein synthesis‐ and PKA‐dependent form of LTP. In keeping with the original nomenclature, we refer to this form of potentiation as LTP2 to distinguish it from the classical form that is independent of these processes (termed LTP1). Pojeong's first contribution was to demonstrate that CP‐AMPARs specifically mediated LTP2 (Park et al. 2014, 2016). Pojeong then joined the Bristol lab, where he solved the problem regarding the origins of the increase in single channel conductance in LTP by demonstrating that this was specifically due to the insertion of CP‐AMPARs (i.e., LTP2) that is associated with the induction and early expression of LTP2 (Park, Georgiou, et al. 2021). He also found that the ability of synapses to undergo depotentiation was dependent on the form of potentiation (STP, LTP, LTP2) induced (Park, Sanderson, et al. 2019). Working between Bristol and Seoul, he found evidence that CP‐AMPARs mediate both heterosynaptic LTP and heterosynaptic metaplasticity (Park, Kang, et al. 2019, 2021). The latter was a form of synaptic tagging and capture, as discovered by Frey and Morris (Frey and Morris 1997). Pojeong showed that CP‐AMPARs may serve both as the synaptic tag and as a trigger for the generation of plasticity‐related proteins. A study continued in Toronto.

## 7 Toronto (2015–Present Day)

The MRC pulled the plug on the Centre for Synaptic Plasticity in 2012, and around the same time the administrators at the University of Bristol enforced an unhappy merger of preclinical departments and associated consolidation of support staff, all of which impacted my ability to do research effectively. I was attracted to Toronto by its scientific excellence and secured a joint appointment at the University of Toronto and Mount Sinai Hospital. At the University I was appointed Chair of Physiology; a true honor to follow in the footsteps of McLeod and Best and serve in the Department that gave the world insulin. After 3 years, I stood down from this role to assume the Directorship of the Tanz Centre for Research in Neurodegenerative Diseases (CRND). My second position is as a Senior Scientist at the Lunenfeld‐Tannenbaum Research Institute (LTRI) at Mount Sinai Hospital. At the time of writing, these are the two positions that I hold.

Toronto is an amazing cosmopolitan city and the University of Toronto a real powerhouse in neuroscience and medical research (In a recent review conducted by *Nature*, UofT was ranked #2 amongst Universities globally for health sciences research output). To this day I continue to work on NMDARs, AMPARs, mGluRs, and KARs along with associated signaling molecules. I hope to reflect on the Toronto lab's scientific contributions in due course.

## 8 Concluding Remarks

LTP and LTD are the most extensively investigated neuronal phenomena. Unfortunately, the field has been blighted with controversies, but as I discussed in a review co‐authored with Tim Bliss (Bliss and Collingridge 2013), the different interpretations can largely be accounted for by the co‐existence of multiple forms of synaptic plasticity. At CA1 synapses, potentiation involves STP1, STP2, LTP1 and LTP2 (plus LTP3; a transcription‐dependent form of LTP not covered in this article). STP1 through LTP2 overlap in time and are present to varying degrees that depend on the stimulus parameters used to induce and monitor them. These four forms of synaptic potentiation, while all dependent on NMDARs, have different molecular bases. Therefore, unless the forms of plasticity are precisely defined, the interpretation is muddy.

That said, the field has gained huge insights into the complex operations of synapses, with major implications for cognition and numerous disorders of the CNS. Space has limited the number of molecules that I have been able to describe—my focus, given the autobiographical nature of this collection, has been on the molecules that my colleagues and I have studied and some related molecules, included for context. For now, I have presented my current understanding of the mechanisms of hippocampal LTP (Figures 3 and 4) and LTD (Figure 5) based on several decades of research. My ideas are still evolving as new information becomes available, driven by advances in pharmacology, genetics and imaging. Synaptic plasticity is far more complex than the LTP scheme presented in our review of 1993. But far more interesting as a result!

My journey through neuroscience has more than often centered around glutamate receptors. I owe my initial interest in the topic to my undergraduate days at the University of Bristol, where I was taught by Jeff Watkins and Dick Evans, my postgraduate training with John Davies at the School of Pharmacy (now part of UCL) and my first postdoctoral position with Hugh McLennan. The physiological process that I have explored is synaptic plasticity, in particular LTP and LTD in the hippocampus, inspired by the classic paper of Bliss & Lomo (Bliss 2025). I consider that the discovery of L‐glutamate as the major (excitatory) neurotransmitter in the mammalian CNS and the discovery of LTP to be two of the most important discoveries in the entirety of biology. Together they explain much of what defines us as human individuals, forming the basis of learning and memory and other cognitive functions in vertebrates. Errors in plasticity at glutamate synapses underlie, or at least contribute to, many major brain disorders, making the topic of great interest for understanding the etiology of disease and for designing therapeutic strategies. Working at the interface of these two major fields and getting to know these, and other, pioneers of glutamate receptors and synaptic plasticity has been an amazing experience—beyond one that I can put adequately into words. Writing this article has tested my memory—I hope my NMDA receptors were working well as I laid down the memories. If I have made factual errors or major omissions, blame my NMDARs and synaptic plasticity—after all, they are only human!

## Data Availability

Data sharing not applicable to this article as no datasets were generated or analysed during the current study.
